# Effects of turmeric on reducing heterocyclic aromatic amines in Chinese tradition braised meat products and the underlying mechanism

**DOI:** 10.1002/fsn3.2518

**Published:** 2021-08-18

**Authors:** Qiang Wang, Jin Li, Kaikai Li, Chunmei Li

**Affiliations:** ^1^ College of Food Science and Technology Huazhong Agricultural University Wuhan China; ^2^ Key Laboratory of Environment Correlative Food Science Ministry of Education Huazhong Agricultural University Wuhan China

**Keywords:** 1,2,3,4‐Tetrahydro‐β‐carboline‐3‐carboxylic acid, Curcumin, Turmeric, β‐carboline heterocyclic amines

## Abstract

Braised meat products are kinds of popular traditional meat food in China. However, current data on the formation of Amino‐carboline congeners Heterocyclic aromatic amines (HAAs) and the inhibitory methods in braised meat products are limited. In the present study, the inhibition effect of turmeric and curcumin on the formation of β‐carboline heterocyclic aromatic amines in braised meat were investigated. And the preliminary mechanism of curcumin inhibiting the formation of β‐carboline heterocyclic amines was also explored in the chemical model. The results indicated that 5% of turmeric could significantly inhibit the formation of harman (94.8%) and norharman (49.56%) in braised meat, and curcumin was one of the key active compound accounting for this effect. In the tryptophan model, 0.05 mmol of curcumin significantly inhibited the formation of norharman and harman by over 70% (*p* < .05). Further investigation indicated that curcumin inhibited the formation of β‐carboline heterocyclic amines mainly by inhibiting the formation of carbonyl compounds and 1,2,3,4‐Tetrahydro‐β‐carboline‐3‐carboxylic acid and scavenging β‐carboline HAAs. These results could provide a natural spice‐based method for reducing heterocyclic aromatic amines in Chinese tradition braised meat products.

## Graphical abstract

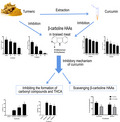

**PRACTICAL APPLICATION**

The present study aimed to explore the material basis of turmeric being responsible for the Heterocyclic aromatic amines (HAAs) inhibition and their mechanisms. These results could provide scientific basis for a natural spice‐based method for reducing HAAs in Chinese tradition braised meat products.

## 1 INTRODUCTION

Heterocyclic aromatic amines (HAAs) are carcinogenic and mutagenic substances produced during the high‐temperature processing of higsh protein meat and commonly found in high‐temperature meat products such as frying and barbecue (Adamson et al., 2010; Jinap et al., 2015). Until now, more than 25 kinds of HAAs have been identified, and they could be divided into two categories according to the formation process: Aminoimidazo azaarenes and Amino‐carboline congeners (Cheng et al., 2010; Oz & Cakmak, 2016). In previous studies, most researches focused on the formation of HAAs in high‐temperature processed meat products such as fried and grilled foods. By contrast, limited data on HAAs were available in braised meat products, which are a very popular meat food in China and other Asian countries. Compared with the high‐temperature processed meat products, the processing temperature of braised meat is lower (usually under 100°C), but the processing time is longer. Moreover, in traditional braised meat production, brine (also known as aged brine) is often used multiple times. Therefore, a large amount of related harmful HAAs substances, such as harman and norharman, may be produced during this repeated and prolonged heating process. Harman and norharman belong to β‐carboline heterocyclic amines, which are two main HAAs in braised meat (Li et al., 2019). For example, Pan found that the contents of norharman and harman in braised mutton were 6.456 and 7.050 ng/g, respectively (Pan et al., 2014). Epidemiological studies indicated an association between HAAs and cancer, particularly colon, rectal, breast, and prostate (Adeyeye, 2020). Although harman and norharman do not show no mutagenesis in the Ames/Salmonella experiment, they are potential co‐mutagenic compounds that may not only enhance the toxicity of other HAAs, but may also increase the risk of cancer (Cheng et al., 2010; Vidal et al., 2020; Zhang et al., 2020). Therefore, it is of great significance to restrain the formation of HAAs in the production process of braised meat.

Formation of heterocyclic amines is a complex process with many important substrates as precursors. It has been found that tryptophan was an important substrate of β‐carboline heterocyclic amines and glucose promotes the formation of β‐carboline HAAs (Pfau & Skog, 2004). Herraiz found that tetrahydro‐β‐carboline (THβC) was one precursor of norharman and harman in chemical models (Herraiz, 2000a). In addition, 1,2,3,4‐Tetrahydro‐β‐carboline‐3‐carboxylic acid (THCA) and 1‐methyl‐1,2,3,4‐tetrahydro‐β‐carboline‐3‐carboxylic acid (MTCA) are also the precursors of β‐carboline HAAs (Herraiz, 2000b). The contents of THCA, MTCA and β‐carboline (norharman and harman) gradually increased with the prolongation of cooking time of fish and meat, and β‐carboline could be formed by THβC oxidation (Herraiz, 2000b). Previous studies have indicated that Pictet‐Spengler reaction was an important pathway for the formation of β‐carboline HAAs. Precursors such as tryptophan undergo Pictet‐Spengler reaction to form THβC, which was further oxidized and decarboxylated to form norharman and harman (Herraiz & Ough, 1993). More importantly, free radical reactions are also involved in the formation of heterocyclic amines (Kikugawa, 1999), and some antioxidants or plant extracts can scavenge the free radicals such as pyridine or pyrazine produced in Maillard reaction, thus inhibiting the formation of heterocyclic amines (Chen et al., 2014; Lu et al., 2011).

Many factors that affect the production of HAAs in food process, including cooking method, external additives, and food type (Adeyeye & Ashaolu, 2021; Dong et al., 2020). In recent years, the antioxidant and HAAs inhibition activities of plant extracts and spices have attracted much attention of researchers. For example, grape seed extract could decrease the levels of total HAAs in charcoal‐barbecued beef meatballs (65%) and oven roasted chicken meatballs (37%) (Keskekoglu & Uren, 2017). Blueberry, raspberry, and strawberry extracts could reduce the formation of carcinogenic heterocyclic amines in fried camel, beef and chicken meats (Khan et al., 2020). Compared with these plant extracts, spices could not only inhibit the producing of HAAs, but also enhance the flavor and taste of food. Our previous study found that the traditional Chinese spice ginger showed effective inhibition on the formation of HAAs in braised chicken (Li et al., 2019). Considering turmeric belongs to the ginger plant family and had higher content of curcumin than ginger, and it has been used as a traditional medicinal plant and spice for a long time in Asia, we supposed that turmeric might be a be a more potential spice in reducing HAAs in Chinese traditional braised meat products than ginger. Therefore, the effect of turmeric on the formation of HAAs in braised chicken were evaluated, and the material basis of turmeric accounting for the HAAs inhibition and their mechanisms were also explored. We believed that our results could provide scientific basis for a natural spice‐based method for restraining heterocyclic aromatic amines in Chinese tradition braised meat products.

## 2 MATERIALS AND METHODS

### 2.1 Chemicals and materials

Chicken breast (recessive white chicken) and turmeric were purchased from the local supermarket (Wuhan, Hubei, China). Norharman and harman were obtained from Toronto Research Chemicals Inc. (Downsview, Ont., Canada). Curcumin, tryptophan, phenylalanine, and creatine were purchased from Shanghai yuanye Bio‐Technology Co., Ltd (Shanghai, China). Methanol and acetonitrile (High Performance Liquid Chromatography (HPLC) grade) were purchased from Thermo Fisher Scientific (Waltham, MA, USA). Acetic acid, ammonium acetate, sodium hydroxide, ammonium hydroxide, dichloromethane, diethylene glycol, and hydrochloric acid glucose were purchased from Sinopharm Chemical Reagent Co., Ltd. (Shanghai, China). The MCX SPE column (Oasis® MCX 3cc, 60 mg, Extraction Cartridges) was obtained from Waters Corporation (Waters, Milford, MA, USA).

### 2.2 Cooking of the braised meat

Forty gram of chicken breast was cut into small pieces (4 cm × 2 cm × 1 cm), and then, 200 ml of water, 1 g of sugar, 1 g of salt, and 5 ml of soy sauce were added. Followed by heating at boiling temperature for 1 h. The chicken breast was then collected and dried at 45℃ for 4 h to make minced meat and then kept at −20℃.

### 2.3 Effect of ginger, turmeric, and curcumin on the production of β‐carboline heterocyclic amines in the sauce braised meat

Ginger and turmeric powder were added to chicken at levels of 1%, 3%, and 5% (w/w), and curcumin was added to chicken at concentrations of 0.03, 0.05, and 0.07 mmol related to the mass of chicken. The heating processing was the same as described in 2.2. After being braised, the chicken breast was collected and dried to make minced meat and then kept at −20℃ for further study. The operation was repeated three times to collect the chicken. The contents of HAAs were determined according to the method described in Section 2.9.

### 2.4 Effect of curcumin on the formation of β‐carboline heterocyclic amines in chemical model

In brief, the compositions of chemical model for generating β‐carboline heterocyclic amines were 0.4 mmol of tryptophan and 10 ml of 75% diethylene glycol (Chen & Meng, 1999). 0.4 mmol of tryptophan and 0.05 mmol of curcumin were added to 10 ml of 75% diethylene glycol, after heating the mixture at 100℃ by the electric constant temperature blast drying oven for 30, 45, 60, 75, and 90 min, the mixture was immediately cooled in ice water for 10 min. Then, the reaction solution was collected and stored in the refrigerator at −20℃.The operation was repeated three times, and the contents of norharman and harman were determined according to the method described in Section 2.9.

### 2.5 Inhibitory effect of curcumin on the formation of THCA in the chemical model

To study the effect of curcumin on the formation of THCA, 0.01, 0.03, and 0.05 mmol of curcumin were added to the above chemical model, respectively, distilled water replaced the curcumin in the blanks. The mixture was heated at 100℃ for 60 min, followed by cooling in ice water immediately. The operation was repeated for three times. Then, the reaction solution was collected and stored in the refrigerator at −20℃. The contents of THCA were determined according to the method described in Section 2.10.

### 2.6 Scavenging effect of curcumin on THCA in the chemical model

The scavenging abilities of curcumin were performed with a pure THCA solution. Briefly, dissolve 10 mg of THCA in 50 ml of distilled water and collect the supernatant saturated solution. 3 ml of supernatant and 7 ml of distilled water were added to the reaction bottle, and then, different concentrations of curcumin (0.01–0.05 mmol) were added. The samples were vortexed and heated at 100℃ for 60 min and then cooled in ice water. The operation was repeated for three times. The contents of THCA were determined according to the method described in Section 2.10.

### 2.7 Effect of curcumin on the formation of carbonyl compounds in the chemical model

To investigate the inhibitory ability of curcumin on the formation of carbonyl compounds, 300 μl of reaction solution (0.4 mmol of tryptophan in 10 ml of 75% diethylene glycol) were mixed with 500 μl of 10 mM 2, 4‐dinitrophenylhydrazine solution (prepared with 2 m hydrochloric acid) and then kept in dark at 30℃ for 60 min. Then, *n*‐hexane was added, vortexed, and centrifuged at 5000 g for 10 min. The *n*‐hexane layer was discarded, and the residual solution was washed with *n*‐hexane for three times to determine the absorbance under 370 nm (Zamora et al., 1997).

### 2.8 Interaction of curcumin with heterocyclic amines

To investigate whether curcumin could directly react with HAAs to reduce their content, 10 ml of HAAs solution (1 mg/ml) was mixed with 0.03 mmol/L of curcumin, and then, 10 ml of diethylene glycol was added into the reaction bottle, vortexed, and kept at 100℃ for 60 min. Distilled water replaced the curcumin in the control group. After the reaction, the bottle was immediately cooled with ice water, and then kept at −20°C for further study. The contents of Norharman and Harman were determined according to the method described in Section 2.9.

### 2.9 The analysis of β‐carboline heterocyclic amines

The assay was carried out according to the method of Gross and Gruter with a minor modification (Gross & Grüter, 1992). 3.0 g of the processed meat, 5 ml of 2 m NaOH solution, and 10 ml of dichloromethane were added in a 50‐ml centrifuge tube, and then, the mixture was vortexed for 10 min and centrifuged at 10,434 × *g* for 10 min. The layer of dichloromethane was transferred to a 50 ml concentrated tube. The operation was repeated twice to collect the eluent.

The MCX SPE column was firstly activated with 3 ml of dichloromethane. All the collected eluent was passed through the column, and the column was eluted with 3 ml of 0.1 mol/L HCl‐methanol (40:60, v/v), 2 ml of methanol, and 2 ml of ultrapure water in turn. Finally, the heterocyclic amines in the column were eluted with a mixture of 3 ml ammonia and methanol (15:85, v/v), then dried at 50℃ with nitrogen, and then redissolved with 400 μl methanol. The methanol solution was passed through the 0.22 μm microporous membrane before HPLC analysis.

HPLC analysis was performed on a Waters high‐performance liquid chromatography according to the method we previously established (Li et al., 2019). Chromatographic column: TSK gel ODS‐80TM (250 mm × 4.6 mm, 5 μm). Mobile phase: 0.05 m ammonium acetate‐acetic acid buffer (A), acetonitrile (B). The flow rate was 1 ml/min. Elution procedure: 0–15 min, 95%−75%(A); 15–25 min, 75%–55% A; 25–30 min, 55%–70% A; 30–35 min, 95% A. The injection volume was 20 μl at room temperature. Norharman and Harman can be detected by programming the fluorescence detector, excitation wavelength/emission wavelength: 300 nm/440 nm. Triplicate analyses were performed for each treatment. The average recoveries of the two HAAs varied between 82.3% and 95.6%, and the relative standard deviation varied between 1.56% and 7.49% (*n* = 3). For norharman, the LOD value was 0.30 (ng/g) and the LOQ value was 0.83 (ng/g). For harman, the LOD value was 0.17 (ng/g) and the LOQ value was 0.33 (ng/g) (Li et al., 2019).

### 2.10 The analysis of THCA

The method of separation, purification, and detection of THCA was according to the method of Herraiz (Herraiz, 2000b) with slight modification. In brief, 3 ml of reaction solution was mixed with 15 ml of 0.6 m HClO4 (including 1 mg/ml semicarbazide) and centrifuged at 4℃ for 15 min (5100 g). The supernatant (5.5 ml) was added to the Waters MCX column. The MCX column was washed with 6 ml of 0.1 m HCl, 2 ml of methanol, 6 ml of distilled water, and 2 ml of 0.4 m phosphate (pH 9.2). Finally, the MCX column was washed with 4 ml of 0.4 m phosphate buffer (pH 9.2): methanol (1:1). The eluate was collected and passed through the 0.22 μm membrane for HPLC analysis (Herraiz, 2000b). HPLC analysis was performed on a Waters 1525 high‐performance liquid chromatography with Waters 2475 fluorescence detector and Waters 717 injector. Separation was carried out on a TSK gel ODS‐80TM (250 mm × 4.6 mm, 5 μm) column. The mobile phase was 0.5 m ammonium acetate‐acetic acid buffer (A)/acetonitrile (B), and elution procedure was as followed: 0–8 min, 90%–68% A; 8–18 min, 68%–10% A; 18–25 min, 10%–90% A; and 25–30 min, 90% A. The flow rate was 1 ml/min with injection volume of 20 μl. Excitation wavelength/emission wavelength were set as 270 nm/343 nm.

### 2.11 Statistics and analysis

Statistical analyses were performed using the SPSS statistical software 25.0 (IBM, Armonk, NY, USA). ANOVA was used to establish significance at *p* < .05.

## 3 RESULTS AND DISCUSSION

**FIGURE 1:**
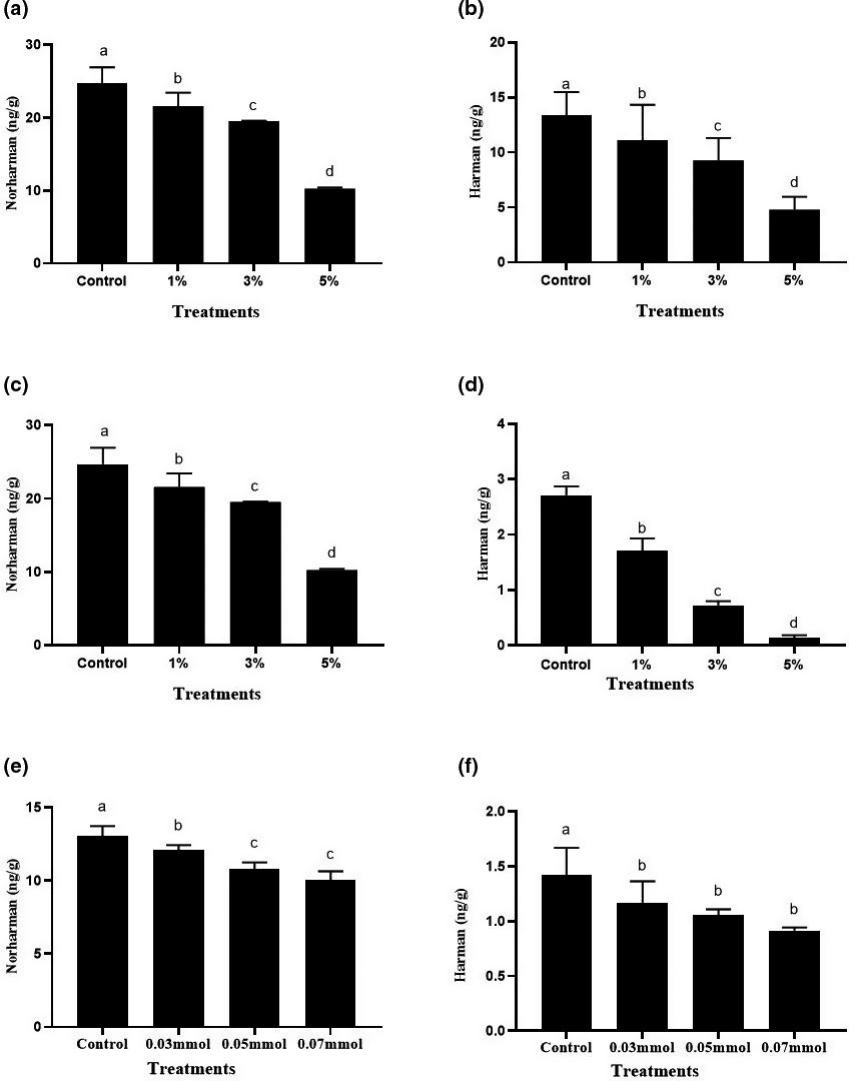
Inhibitory ability of ginger, turmeric, and curcumin on the formation of norharman and harman in braised meat. (a) and (b) Inhibition ability of ginger on the formation of norharman and harman in braised meat. (c) and (d) Inhibition ability of turmeric on the formation of norharman and harman in braised meat. (e) and (f) Inhibition ability of curcumin on the formation of norharman and harman in braised meat. Values marked by different lowercase letters are significantly different (*p* < .05)

Amino‐carboline HAAs, including β‐carboline heterocyclic amines, is generally believed to be formed at temperature higher than 300℃ (Toribio et al., 2002). However, they can also be produced under mild temperature conditions (≤100℃) (Arvidsson et al., 1999). Liao studied the kinetics of the formation of HAAs in the model system and found that norharman and harman have lower activation energy than other polar HAAs, which meant that β‐carboline heterocyclic amines are less sensitive to temperature and are easy to form at low temperature (Liao, 2008). Zhou et al. detected two kinds of HAAs (norharman and harman) in the braised sauce beef and found that the content gradually increased and then stabilized as the cooking temperature increased (Zhou et al., 2021). Shen cooked the braised pork at 100℃ and found that the contents of norharman and harman in braised pork were 84.99 and 140.21 ng/g, respectively (Shen et al., 2018). Amino acids, sugars, and creatine in the meat muscle are important precursors for the formation of HAAs (Adeyeye, 2020). It has been reported that soy sauce contains a large number of amino acids, which are important precursors for the formation of HAAs. Sugar can promote the formation of heterocyclic amines, which may be an important source of β‐carboline heterocyclic amines in sauce stewed meat products (Cheng et al., 2010). In our previous experiments, we found that ginger showed effective inhibition on the formation of HAAs in braised chicken (Li et al., 2019). Considering turmeric also belongs to the ginger plant family and has higher content of bio‐active curcumin than ginger. We hypothesized that turmeric may be a more potential HAAs inhibitory spice than ginger. Therefore, the inhibitory ability of turmeric on the formation of harman and norharman were firstly investigated in traditional braised chicken. As shown in Figure 1, 5% of ginger could inhibit the production of β‐carboline heterocyclic amines by about 60.46%. Turmeric could effectively inhibit the formation of β‐carboline HAAs in braised meat, showing a dose–effect relationship. When the addition amount of turmeric reached 5%, the inhibition rate was 67.12%, which was stronger than that of ginger. According to Li’s report, 5% of dried tangerine peel and cinnamon could inhibit the production of β‐carboline heterocyclic amines by about 33.5% and 41.20%, respectively (Li et al., 2019). Obviously, turmeric showed stronger inhibiting effect than these spices.

It is reported that the addition of nitrites, antioxidants, or spices can inhibit the formation of HAAs in meat products (Adeyeye & Ashaolu 2021). In our previous reports, we also found that antioxidant capacity is strongly associated with inhibition of heterocyclic amine (Li et al., 2019). Considering curcumin is the main antioxidant ingredient of turmeric, we inferred that it may be the material basis accounting for the HAAs inhibition activity of turmeric. Therefore, we further explored the inhibitory effect of curcumin on the formation of HAAs in braised chicken. As shown in Figure 1e and 1f, 0.07 mmol of curcumin (equivalently to 1.28% of turmeric) showed significantly inhibitory activity on the formation of β‐carboline HAAs in braised chicken meat. The contents of harman and norharman were reduced by 35.69% and 23.54%, respectively, which accounted for 96.85% and 94.12% of the inhibition rate of 1% turmeric. Therefore, we inferred that curcumin is the main inhibitory substance of turmeric.

**FIGURE 2:**
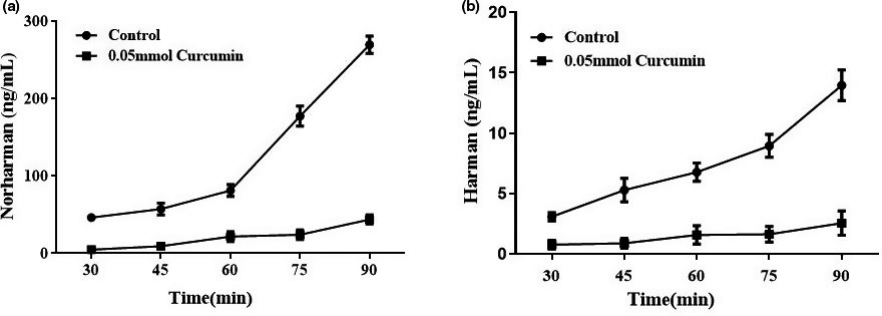
Inhibitory ability of curcumin on the formation of norharman and harman in the chemical model. (a) Diagram of the amount of norharman changing with time. (b) Diagram of the amount of harman changing with time

We further used chemical model to explore the possible mechanisms of curcumin or turmeric on HAAs inhibition. As shown in Figure 2, although the contents of norharman and harman in the model increased with the extension of heating time, curcumin could significantly reverse it. At 90 min, curcumin significantly inhibited the formation of norharman and harman by 83.84% and 81.79%, respectively. This result agreed with that of the braised meat system well, suggesting that curcumin might be the main bio‐activate compounds of turmeric on HAAs inhibition.

**FIGURE 3:**
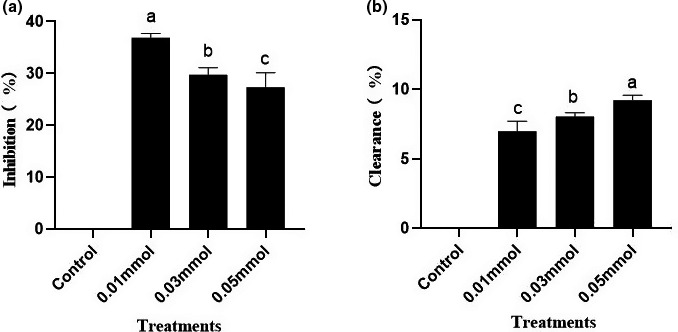
Inhibition and Clearance rate of curcumin on the formation of 1,2,3,4‐Tetrahydro‐β‐carboline‐3‐carboxylic acid (THCA) in the chemical model. (a) Inhibition rate of curcumin on THCA (b) Clearance rate of curcumin on THCA. Values marked by different lowercase letters are significantly different (*p* < .05)

Due to the complex formation mechanism of HAAs, the inhibition mechanism of polyphenols or spice on HAAs is poorly understood. It was confirmed that THCA is an important intermediate for the formation of norharman (Herraiz, 2000a), and therefore, we investigated whether curcumin inhibited the formation of β‐carboline heterocyclic amines by inhibiting the formation of THCA or by clearing it. It can be seen from Figure 3a, curcumin had a strong inhibitory effect on THCA formation, with an inhibition rate of 37.09%, indicating that curcumin could block THCA reaction chain, thus inhibiting the formation of THCA. By contrast, the content of THCA solution did not decrease significantly with the concentration increase of curcumin and the clearance rate was below 10% (Figure 3b), indicating that curcumin inhibited the formation of heterocyclic amines by inhibiting the formation of THCA, not by scavenging it, which was different from the inhibition mechanism of PhIP (Zhou et al., 2017).

**FIGURE 4:**
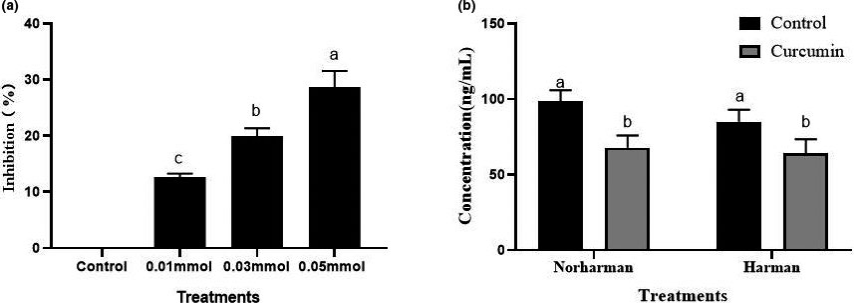
Effect of curcumin on the formation of carbonyl compounds and β‐carboline heterocyclic amines in the chemical model. (a) Inhibition rate of curcumin on the formation of Carbonyl compounds in the chemical model. (b) Scavenging effect of curcumin on norharman and harman in chemical model. Values marked by different lowercase letters are significantly different (*p* < .05)

In addition, carbonyl compounds (including formaldehyde and acetaldehyde) were reported to be important intermediates for the formation of THCA (Totsuka et al., 1999). Therefore, we further investigated the effect of curcumin on inhibiting the formation of carbonyl compounds. It can be seen from Figure 4a that 0.05 mmol of curcumin had a significant inhibitory effect on the formation of carbonyl compounds, with the inhibition rate of 28.70%. Curcumin can reduce the content of carbonyl compounds in the chemical system, thus inhibiting the production of THCA. Yan reported that naringin may could inhibit HAAs formation by removing of THCA intermediates including phenylacetaldehyde, formaldehyde, and acetaldehyde (Yan, 2015). Our results were in line with theirs.

The above results indicated that curcumin could decrease the formation of HAAs. However, whether curcumin could directly clear HAAs to decrease the content was still unclear. Therefore, curcumin was incubated with pure norharman and harman solution to investigate the directly scavenging abilities of curcumin on HAAs. As presented in Figure 4, curcumin could directly react with norharman and harman, thus significantly reducing the content by 31.32% and 24.71%, respectively, suggesting directly clearing norharman and harman was another way of curcumin on decreasing HAAs chemical content.

## 4 CONCLUSION

The results indicated that turmeric could significantly inhibit the formation of harman and norharman in braised chicken, and the key active substances were curcumin. Curcumin could decrease the contents of norharman and harman in in braised chicken products by inhibiting the formation their key intermediates such as THCA and carbonyl compounds and directly clearing them. The results suggested that except for conventional use for providing specific flavor and taste to braised meat products, turmeric could also exert strong potential to reduce the content of β‐carboline HAAs in heat‐processed meat products which highlighted a new way of reducing HAAs in Chinese tradition braised meat products by turmeric.

## CONFLICT OF INTEREST

The authors declare that they do not have any conflict of interest.

## ETHICAL APPROVAL

This study does not involve any human or animal testing. Written informed consent was obtained from all study participants.
